# *Tosanoides obama*, a new basslet (Perciformes, Percoidei, Serranidae) from deep coral reefs in the Northwestern Hawaiian Islands

**DOI:** 10.3897/zookeys.641.11500

**Published:** 2016-12-21

**Authors:** Richard L. Pyle, Brian D. Greene, Randall K. Kosaki

**Affiliations:** 1 Bernice P. Bishop Museum, 1525 Bernice Street, Honolulu, Hawai‘i 96817, USA; 2 Association for Marine Exploration, 4075A Koko Dr., Honolulu, Hawai‘i 96816; 3 NOAA Papahānaumokuākea Marine National Monument, 1845 Wasp Blvd, Building 176, Honolulu, Hawai‘i 96818, USA

**Keywords:** Mesophotic Coral Ecosystem, Closed-Circuit Rebreather, Endemic, Papahānaumokuākea Marine National Monument, President Obama

## Abstract

The new species *Tosanoides obama* is described from two specimens collected at a depth of 90–92 m off Kure Atoll and Pearl and Hermes Atoll, Northwestern Hawaiian Islands. It differs from the other two species of this genus in life color and in certain morphological characters, such as number of pored lateral-line scales, pectoral-fin rays, snout length, anterior three dorsal-fin spine lengths, dorsal-fin profile, and other characters. There are also substantial genetic differences from the other two species of *Tosanoides* (d ≈ 0.10 in mtDNA cytochrome oxidase I). The species is presently known only from the Northwestern Hawaiian Islands within the Papahānaumokuākea Marine National Monument.

## Introduction

Since 2009, the National Oceanographic and Atmospheric Administration (NOAA) has conducted annual research cruises to the Northwestern Hawaiian Islands led by the third author (RKK) involving advanced mixed-gas diving operations to explore and document mesophotic coral ecosystems (MCEs; coral-reef habitat at depths of 30–150 m) within the Papahānaumokuākea Marine National Monument. In addition to conducting quantitative surveys of fishes (e.g., Kane et al. 2014, Kosaki et al. 2016), exploratory deep dives have focused on documenting species occurrence records in an effort to develop comprehensive checklists of fishes from each major reef and island within the Monument. During one such deep dive on 5 June 2016 off Kure Atoll, the first author (RLP) observed a small pink fish that at first appeared to be a juvenile *Pseudanthias thompsoni* (Fowler, 1923), but had a prominent red spot on the posterior portion of the dorsal fin. After capturing a brief video clip of this fish, as well as a second similar individual that lacked the spot on the dorsal fin (YouTube 2016), the specimen with the spot was collected alive. The following day, the second author (BDG) observed a group of three individuals of the same fish (one with the red spot on the dorsal fin, and two without) at a depth of 90 m off Pearl and Hermes Atoll, and collected one of the individuals (lacking the spot) alive.

The specimens represent an undescribed species within the serranid subfamily Anthiadinae Poey, 1861 (commonly spelled Anthiinae, but see van der Laan et al. 2014, 2016 and *Discussion* section below), within the genus *Tosanoides* Kamohara, 1953. The genus currently includes two nominal species, both considered valid and both from the tropical and subtropical northwestern Pacific: *Tosanoides filamentosus* Kamohara, 1953 (type species), and *Tosanoides flavofasciatus* Katayama & Masuda, 1980. Herein we describe the third member of the genus, *Tosanoides obama*, based on morphologic and genetic differences compared with the other two known species.

## Methods

Specimens were collected with hand nets during deep dives using mixed-gas, closed-circuit rebreathers.

Standard length (SL) was measured from the tip of the snout to the caudal-fin base. Total length (TL) was measured from the tip of the snout to the posterior edge of the filamentous extensions on the caudal fin. Head length was measured from the tip of the snout to the posterior-most edge of the fleshy flap near the upper end of the gill opening. Body depth is the greatest depth of body measured as a vertical from the ventral edge of the abdomen to the upper edge of scaled fleshy sheath of the dorsal fin (typically from about fourth or fifth dorsal spine). Width of the body is the maximum width. Snout length is the distance from the tip of the snout to the closest point on the bony orbit. Predorsal length is the distance from the tip of the snout to the angle formed by the scaled fleshy sheath at the insertion point of the first dorsal-fin spine, when erected. Preanal length is the distance from the tip of the snout to angle formed by the scaled fleshy sheath at the insertion point of the first anal-fin spine, when erected. The base of the dorsal fin is measured from the extreme base of the first dorsal-fin spine to the extreme base of the last dorsal-fin soft ray. The base of the anal fin is measured from the extreme base of the first anal-fin spine to the extreme base of the last anal-fin soft ray. Orbit diameter is the maximum diameter of the bony orbit. Interorbital width is the width of the bony interorbital space. Depth of the caudal peduncle is the least depth. Pelvic-fin spine length was measured from the extreme base of the pelvic-fin spine to its distal tip. Pelvic fin length is the length of the first ray from its extreme base to the distal tip of the filamentous extension. Length of spines and soft rays of dorsal and anal fins were measured from the extreme base to the most distal tip. Caudal-fin length is defined as the difference between TL and SL. Pectoral-fin length was measured as the longest fin ray, from its extreme base to its tip.

The last dorsal- and anal-fin soft rays are branched to the base and were counted as a single ray. Caudal-fin ray counts include small unsegmented and rudimentary rays. Pectoral-fin ray counts include first two and last two unsegmented and rudimentary rays. Lateral-line scale counts include only those scales with pores. Scale row counts above and below lateral line to origins of dorsal and anal fins (respectively) include small truncate scales at bases of respective fins. Vertebral counts include the first vertebra fused to the skull, and the last vertebra fused to the hypural plate.

All counts and measurements except vertebrae were made directly from specimens. Measurements were made using dial calipers with +/- 0.05 mm precision. Lengths of dorsal- and anal-fin spines and soft rays were made with the aid of a bright light transmitted from behind the fins to reveal the position of their extreme bases. Gill-raker count for the holotype was obtained by removing the first gill arch from the right side of the specimen (not counted on the paratype). Vertebral counts were made from x-radiographs.

Head length, depth of body, width of body, snout length, predorsal length, preanal length, length of dorsal-fin and anal-fin bases, orbit diameter, interorbital width, caudal-peduncle depth, and lengths of fin spines and rays are expressed as percent of SL. Counts and measurements for the paratype, if different from the holotype, are presented in parentheses after the value for the holotype.

Description template and wording modified from Katayama and Masuda (1980) for consistency.

The holotype has been deposited at the Bernice Pauahi Bishop Museum fish collection, Honolulu (BPBM), and the paratype has been deposited at the U.S. National Museum of Natural History, Washington, D.C. (USNM).

Fresh tissue samples were obtained from the holotype and paratype. DNA barcodes (cytochrome c oxidase I; COI) were sequenced following the protocol described in Copus et al. (2015). Barcode of Life Database (BOLD) identifiers for DNA sequences are presented along with museum catalog numbers for type material and non-type specimens.

## Taxonomy

### Tosanoides obama

Taxon classificationAnimaliaPerciformesSerranidae

Pyle, Greene & Kosaki sp. n.

http://zoobank.org/18C72D73-00C3-40E4-B27F-FA7748A1251E

**Figure 1.**
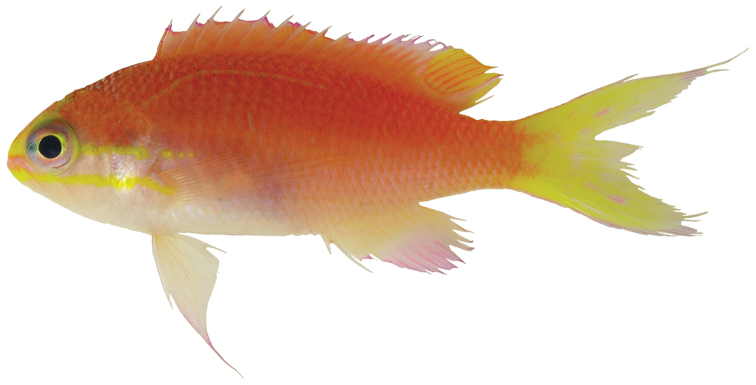
Holotype of *Tosanoides obama* (BPBM 41315), collected at a depth of 90 m off Kure Atoll, Northwestern Hawaiian Islands. Photo by R. L. Pyle.

**Figure 2.**
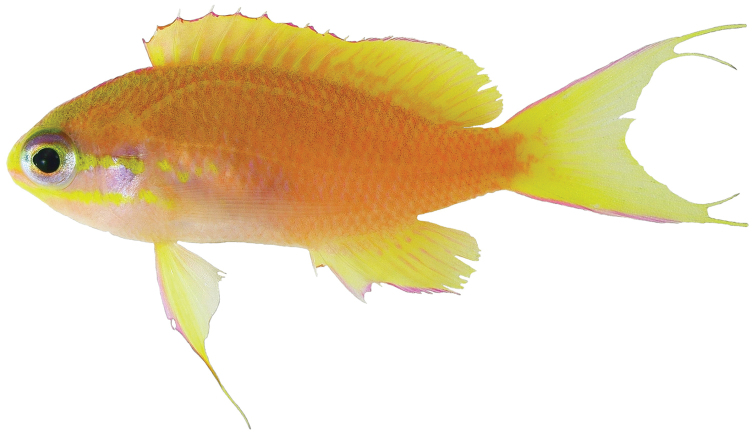
Paratype of *Tosanoides obama* (USNM 440451), collected at a depth of 92 m off Pearl and Hermes Atoll, Northwestern Hawaiian Islands. Photo by R. L. Pyle.

**Figure 3.**
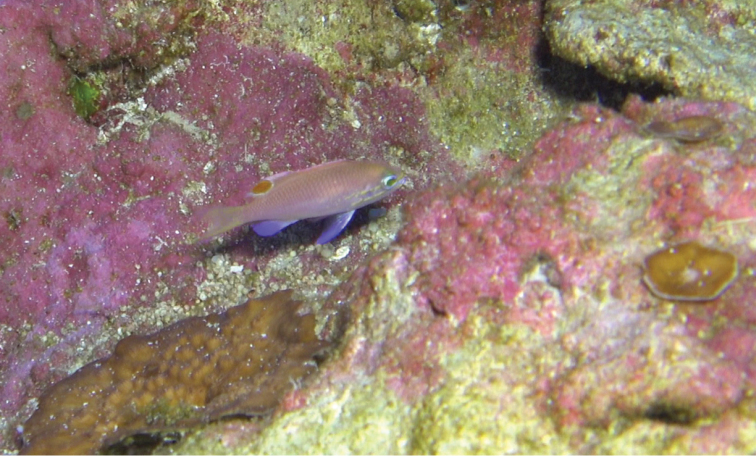
Holotype of *Tosanoides obama* immediately prior to collection, at a depth of 90 m off Kure Atoll, Northwestern Hawaiian Islands. Cropped frame from video by R. L. Pyle.

**Figure 4.**
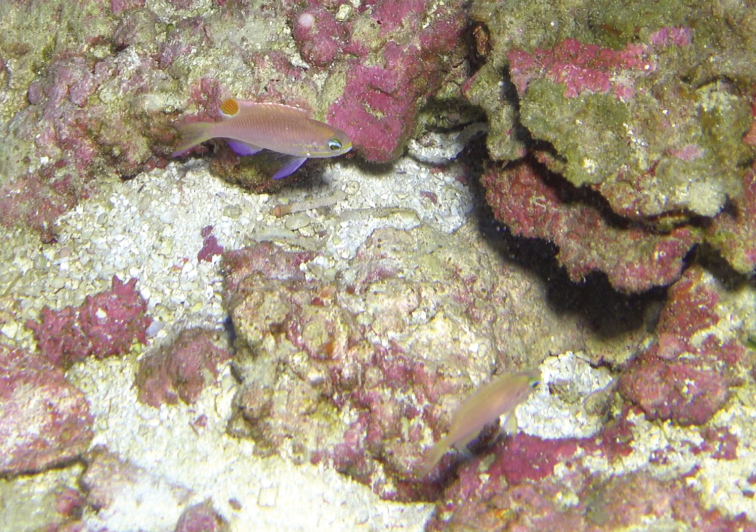
Holotype of *Tosanoides obama* (upper left) alongside presumed female (lower right, not collected) immediately prior to collection of the holotype, at a depth of 90 m off Kure Atoll, Northwestern Hawaiian Islands. Both fish retreated into the same hole moments after this image was captured. Cropped frame from video by R. L. Pyle.

**Figure 5.**
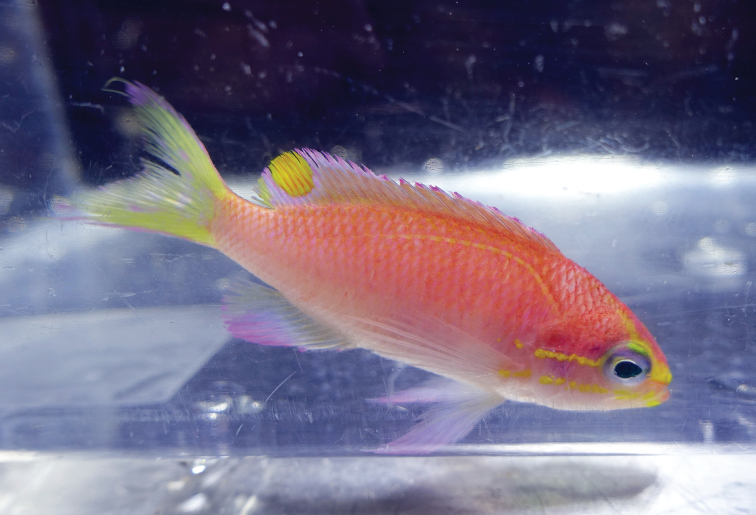
Holotype of *Tosanoides obama* shortly after collection, alive in a holding tank aboard the NOAA Ship *Hi’ialakai*. Photo by R. L. Pyle.

**Figure 6.**
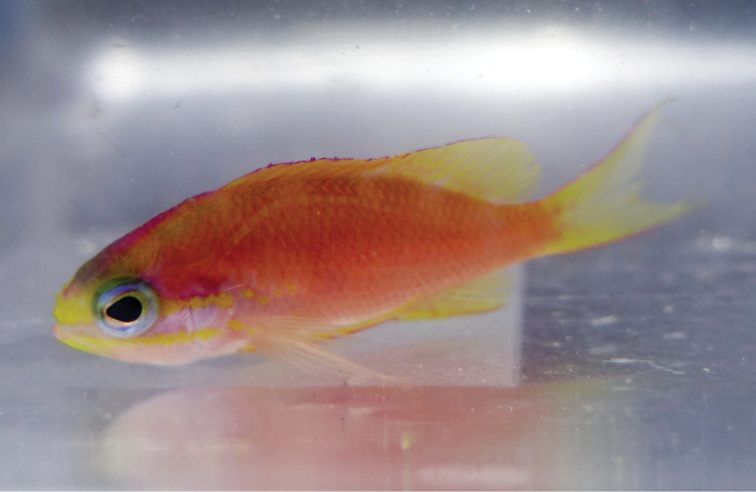
Paratype of *Tosanoides obama* shortly after collection, alive in a holding tank aboard the NOAA Ship *Hi’ialakai*. Photo by R. L. Pyle

Figs 1 , 2 , 3 , 4 , 5 , 6

#### Type locality.

Northwestern Hawaiian Islands, Kure Atoll, north side, 28.4918°N, 178.2879°W.

#### Holotype.

BPBM 41315, male, Barcode of Life TOSOB001-16 (submitted to GenBank), 43.2 mm SL, Northwestern Hawaiian Islands, Kure Atoll, north side, 28.4918°N, 178.2879°W, 90 m, 5 June 2016, R. L. Pyle, aboard NOAA ship *Hi‘ialakai* (Cruise: HA-16-04), hand nets, limestone bottom with small holes. Found in association with a single presumed female (not collected).

#### Paratype.

USNM 440451, immature, Barcode of Life TOSOB002-16 (submitted to GenBank), 28.4 mm SL, Northwestern Hawaiian Islands, Pearl and Hermes Atoll, northwest side, 27.9095°N, 175.9359°W, 92 m, 6 June 2016, B. D. Greene, aboard NOAA ship *Hi‘ialakai* (Cruise: HA-16-04), hand nets, limestone bottom with small holes. Found in association with two other individuals, one presumed male and one presumed female (not collected).

#### Diagnosis.

A species of *Tosanoides* (*sensu* Katayama & Masuda, 1980) distinguished by the following combination of characters: fourth or fifth dorsal spine the longest, dorsal-fin soft rays 17; anal-fin soft rays 8; pored lateral-line scales 33 or 34; head 2.9–3.0 in SL; body depth 2.8–2.9 in SL; color in life pink or yellowish pink on head and body, slightly darker dorsally fading ventrally; snout and region immediately dorsal to eye bright yellow, with a thin bright yellow band extending dorsally on either side of nape; a thin bright yellow horizontal stripe extending horizontally from posterior middle edge of eye posteriorly across most of operculum, continuing as a series of irregular oblong spots on midline of body from just posterior to gill opening to a point approximately one-fourth to one-half of pectoral fin; a second thin bright yellow stripe extending posteriorly from lower jaw across maxilla just ventral to eye and continuing horizontally across operculum and base of pectoral fin; dorsal fin pink or yellowish pink with darker pink regions on membranes, and a bright magenta margin extending from tip of first dorsal fin posteriorly on anterior half of soft dorsal fin; males with a large circular ocellate spot covering posterior one-third of soft dorsal fin, bluish magenta on perimeter and dark red with faint yellow stripes centrally; anal and pelvic fins magenta or yellow; caudal fin translucent yellow, more pale and translucent medially and distally, with bright magenta margins extending along margins of both lobes.

#### Description.

Dorsal fin X,17, last soft ray branched to base; anal fin III,8, last soft ray branched to base; pectoral-fin rays 14; pelvic-fin rays I,5; principal branched caudal rays 14, upper procurrent unbranched caudal rays 6, lower procurrent unbranched caudal rays 4; pored lateral-line scales 33 (34); scale rows above lateral line to origin of dorsal fin 3 (4); scale rows below lateral line to origin of anal fin 14 (13); gill rakers on upper limb 10, on lower limb 22; vertebrae 26 (10+16).

Body ovoid, compressed, its greatest depth 2.88 (2.84) in SL, the width just posterior to gill opening 2.00 (2.50) in depth; head length 2.88 (2.96) in SL; snout short, its length 7.14 (6.40) in head; orbit diameter 2.88 (2.67) in head; interorbital convex, the least bony width 3.57 (3.43) in head; least depth of caudal peduncle 2.83 (2.59) in head.

Mouth large, oblique and protractile; lower jaw not projecting beyond the upper when mouth closed; maxilla 2.08 (2.04) in head, expanded distally, reaching below posterior border of pupil, slightly diagonal, the gape forming an angle of about 20° to the horizontal, supramaxilla absent. A pair of nostrils on either side of head, close together, directly in front of eye, anterior nostril in a membranous tube with an elevated posterior edge, posterior nostril with a slight rim anteriorly. Teeth in upper jaw villiform, forming a band broader anteriorly with a pair of canines on each side and another pair of canines slightly posteriorly and internally directed backward, an outer row of about 10 slender canines on each side of jaw curved forward; lower jaw with a patch of villiform teeth anteriorly; one canine on each side anteriorly facing forward and a second canine on each side curved forward, an outer row of about 15 slender canines like those of the upper jaw, posterior ones pointing forward; small teeth on vomer and palatines, teeth on vomer in a triangular band; tongue pointed, smooth. Preopercle with a round angle, upper limb serrate with about 25 spinules, lower limb smooth; opercle with two flat spines, upper one longest and at apex; subopercle and interopercle smooth. Gill rakers long and numerous, with 10 rakers on the upper limb and 22 on the lower limb, longest raker much longer than gill filament.

Dorsal fin very slightly notched, inserted slightly posterior to dorsal end of gill opening, its base 1.76 (1.67) in SL; first dorsal-fin spine 4.29 (4.17) in head, second dorsal-fin spine 3.33 (3.43) in head, third dorsal-fin spine 3.06 (2.91) in head, fourth dorsal-fin spine the longest, 2.68 (2.40) in head, fifth dorsal-fin spine 2.88 (2.40) in head, last dorsal-fin spine 3.06 (2.67) in head; membranes between anterior dorsal-fin spines mildly incised, progressively less so posteriorly; longest dorsal soft ray (seventh or eighth) 1.32 (1.92) in head. Anal fin originating below base of second dorsal soft ray; second anal spine slightly stronger than the third; length of first anal-fin spine 5.77 (4.80) in head, second anal-fin spine 2.54 (1.96) in head, third anal-fin spine 2.50 (1.85) in head; posterior margin of anal fin rounded; length of longest anal soft ray (fifth or sixth) 1.70 (1.48) in head. Pectoral fins subsymmetrical, longer than head, reaching a vertical at base of third anal soft ray, their length 2.63 (2.49) in SL; caudal fin deeply convex, upper and lower lobes each with two filamentous extensions on their outermost principle rays; pelvic spine 2.00 (1.88) in head; first soft ray of pelvic fin with a filamentous extension (broken in holotype), its length (1.67), in SL.

Scales moderately large, ctenoid; 3 (4) in a series from origin of dorsal fin to lateral line, 14 (13) from origin of anal fin to lateral line; head closely scaled except for lips and tip of snout anterior to nostrils; dorsal and anal fins with small scales basally, a single row on spinous portion of dorsal fin, reaching distally about 1/5 of distance to outer margin posteriorly on soft portions of dorsal and anal fins; about 7 or 8 vertical scale rows on base of caudal fin; scales on pectoral fins basally, extending posteriorly on lower half of pectoral fin approximately one third distance to posterior margin. Lateral line high, concurrent with back, forming an angle below last several dorsal rays and extending along middle of caudal peduncle to base of caudal fin.

Color in life as in Figures 1–6. This species is sexually dichromatic. Male (holotype; Figures 1, 3–5): body and head pink, slightly darker dorsally fading ventrally; snout and region immediately dorsal to eye bright yellow, with a thin bright yellow band extending dorsally on either side of nape from dorsal edge of eye to about one-third to one-half distance to origin of dorsal fin; nape pink; a thin bright yellow stripe extending horizontally from posterior middle edge of eye across most of operculum, continuing as a series of irregular oblong spots on midline of body from just posterior to gill opening to a point approximately one-fourth to one-third of pectoral-fin length; a second thin bright yellow stripe extending posteriorly from lower jaw across maxilla just ventral to eye and continuing horizontally across operculum and base of pectoral fin; a very thin faint yellow stripe along anterior two thirds of lateral line; dorsal fin pink with a bright magenta margin extending from tip of first dorsal-fin spine to anterior half of soft dorsal fin, a large circular ocellate spot covering posterior one-third of soft dorsal fin, extending from base of fin to outer margin, broadly bluish magenta on perimeter and dark red with faint yellow stripes centrally; anal fin magenta except for anterior base; pelvic fins translucent magenta with a pale blue pelvic spine; caudal fin translucent yellow, paler and translucent medially and distally, with bright magenta margins extending along dorsal and ventral margins; live male held under duress in captivity (Figure 5) paler pink over most of body, with faded coloration on median fins and pelvic fins, central portion of ocellate spot bright yellow with thin red lines corresponding to soft dorsal-fin rays. Immature (paratype, Figures 2, 6) and presumed female (Figure 4) fish: body and head yellowish pink, fading ventrally on abdomen and chest; snout and region immediately dorsal to eye bright yellow, with a dusky yellow band extending dorsally on either side of nape from dorsal edge of eye to origin of dorsal fin; nape with a magenta stripe extending from a point just dorsal to snout horizontal to midline of eye and extending dorsally along nape, tapering to a point at origin of dorsal fin; a thin bright yellow stripe extending horizontally from posterior middle edge of eye across most of operculum, continuing as a series of irregular oblong spots on midline of body from just posterior to gill opening to a point approximately one-third to one-half of pectoral-fin length; a second thin bright yellow stripe extending posteriorly from lower jaw across maxilla just ventral to eye and continuing horizontally across operculum and base of pectoral fin; dorsal fin yellowish pink with darker pink regions on membranes between dorsal-fin spines and basally on soft portion of fin; a bright magenta margin extending from tip of first dorsal-fin spine to anterior half of soft dorsal fin; anal fin yellow with pink blotches; pelvic fins translucent yellow anteriorly, white posteriorly, spine bright magenta; caudal fin translucent yellow, paler and translucent medially and distally, with bright magenta dorsal and ventral margins; live immature fish held under duress in captivity (Figure 6) darker pink over most of body.

Color in alcohol uniformly pale yellow except for eye, which is black.

Morphometric data for selected characters of type specimens are provided in Table 1.

**Table 1.** Morphometric and meristic data for selected characters of type specimens of *Tosanoides obama*. Values of morphometric data (other than TL and SL) are represented as % of SL.

	Holotype	Paratype
**Morphometrics**	BPBM 41315	USNM 440451
Sex	Male	Immature
Total length (TL) in mm	61.5	41.0
Standard length (SL) in mm	43.2	28.4
Head length	35	34
Body depth	35	35
Body width	17	14
Snout length	4.9	5.3
Predorsal length	34	36
Preanal length	65	62
Base of dorsal fin	57	60
Base of anal fin	19	21
Orbit diameter	12	13
Interorbital Width	10	10
Caudal Peduncle Depth	12	13
Pelvic Spine	17	18
Pelvic Fin	32	60
First Dorsal Spine length	8.1	8.1
Second Dorsal Spine length	10	10
Third Dorsal Spine length	11	12
Fourth Dorsal Spine length	13	14
Fifth Dorsal Spine length	12	14
Last Dorsal Spine length	11	13
Longest Dorsal Ray length	26	18
First Anal Spine length	6.0	7.0
Second Anal Spine length	14	17
Third Anal Spine length	14	18
Longest anal ray length	20	23
Caudal fin length	42	44
Pectoral fin length	38	40
**Meristics**		
Dorsal Spines	X	X
Dorsal rays	17	17
Anal Spines	III	III
Anal Rays	8	8
Pectoral Rays	14	14
Caudal Rays	6+7+7+4	6+7+7+4
Pored lateral line scales	33	34
Dorsal scale rows	3	4
Ventral scale rows	14	13
Gill rakers	10+22	-

#### Distribution.

*Tosanoides obama* is known on the basis of two specimens and three additional individuals observed at a depth of 90–92 m at Kure Atoll and Pearl and Hermes Atoll, within the Northwestern Hawaiian Islands. Gooding (1980) listed a single individual of *Tosanoides filamentosus* among the fishes collected during a series of trawls at depths of 140–170 fathoms (256–311 m) in 1975–1979 at Hancock Seamount (located approximately 360 km northwest of Kure Atoll). This record appears to represent the only basis for subsequent reports of this species in or near the Hawaiian Archipelago (e.g., Humphreys et al. 1984, Uchida and Uchiyama 1986, Mundy 2005, Hart and Pearson 2011). Unfortunately, the fish reported by Gooding was not preserved, therefore there is no way to determine whether it was correctly identified as *Tosanoides filamentosus*, or perhaps was an individual of *Tosanoides obama*, or a different species.

*Tosanoides obama* is the only coral-reef fish species endemic to the Papahānaumokuākea Marine National Monument (which includes part of Hancock Seamount), although further exploration of MCEs in nearby regions may yet reveal its presence elsewhere. This is consistent with the observation that fish assemblages on deep coral reefs have proportionally more endemic species than on shallow reefs (Pyle 1996, Kane et al. 2014, Kosaki et al. 2016).

#### Habitat and ecology.

Two groups of *Tosanoides obama* have been observed in nature; one consisting of two individuals (the male holotype and an apparent female), and the other consisting of three individuals (an apparent male, an apparent female, and the immature paratype). Both groups were found living among small holes in a hard limestone bottom covered with crustose calcareous algae, in areas of apparent ancient shorelines (undercut limestone ledges adjacent to sandy bottom). General habitat characteristics are evident in Figures 3, 4, as well as the videos cited in the bibliography as YouTube (2016). Both sites are characterized by comparatively dense populations of endemic reef-fish species [primarily *Pseudanthias thompsoni* (Fowler, 1923), *Chromis strusakeri* Randall & Swerdloff, 1973, *Caprodon unicolor* Katayama, 1975, and several other endemic labrids, chaetodontids, and pomacanthids]. Quantitative ecological surveys of mesophotic fish assemblages at the northern end of the archipelago indicate that these assemblages are numerically dominated by small-bodied, endemic planktivorous species (Fukunaga et al. 2016). The known habitat and likely trophic habits of *Tosanoides obama* are consistent with this pattern.

#### Etymology.

We name this species *obama* (a noun in apposition) in honor of Barack H. Obama, 44^th^ President of the United States, in recognition of his efforts to protect and preserve the natural environment, particularly through his decision to expand the Papahānaumokuākea Marine National Monument several weeks after the discovery of this new species.

#### Morphological comparisons.

The morphology of this species is consistent with the diagnosis for the genus *Tosanoides* as presented by Katayama and Masuda (1980). Compared with *Pseudanthias* Bleeker, 1871 (the only other genus it resembles), *Tosanoides obama* shares with the other two species of *Tosanoides* fewer pored lateral line scales (30–34, compared with 35–52) number of anal soft rays (8, compared with 6–7), and unbranched pectoral fin rays.

**Figure 7.**
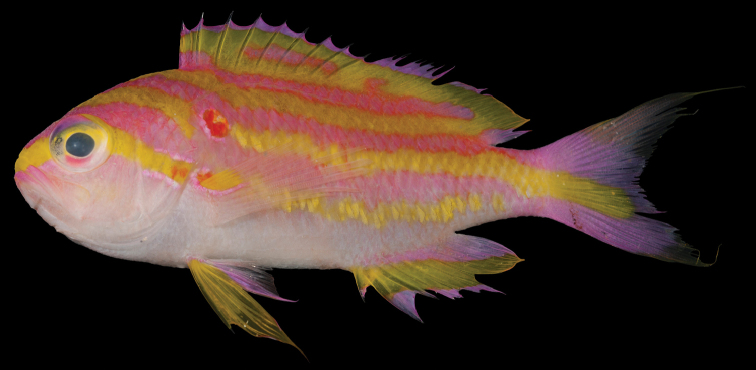
*Tosanoides flavofasciatus*, BPBM 40858, Palau Islands. Photo by R. L. Pyle

*Tosanoides obama* is more similar morphologically to *Tosanoides flavofasciatus* (Figure 7) than to *Tosanoides filamentosus*, primarily on the basis of proportional dorsal-fin spine lengths (third or fourth dorsal spine the longest in *flavofasciatus*, compared with first dorsal spine the longest in *filamentosus*). *Tosanoides obama* differs from both species of *Tosanoides* in number of pored lateral-line scales (33–34, compared with 30–32), number of pectoral-fin rays (14, compared with 13), shorter snout length (6.40–7.14 in head, compared with 4.66–5.86 for *Tosanoides filamentosus* and 2.27–2.89 for *Tosanoides flavofasciatus*), anterior three dorsal-fin spine lengths (4.29–4.17, 3.33–3.43 and 3.06–2.91 in head, compared with 2.03–1.84, 2.24–2.15 and 2.66–2.30 in head for *Tosanoides filamentosus*, and 3.57–3.21, 2.86–2.82 and 2.67–2.52 in head for *Tosanoides flavofasciatus*), and in dorsal-fin profile (slightly notched in *Tosanoides obama*). *Tosanoides obama* also differs from both other *Tosanoides* species in having far less scalation on the median fins (only basally, compared with one half or more of fins), and in the third anal-fin spine (approximately equal to second anal-fin spine, compared with a shorter and less stout third anal-fin spine relative to second anal-fin spine). *Tosanoides obama* additionally differs from *Tosanoides filamentosus* in having a longer anal-fin base (4.81–5.14 in SL, compared with 5.35–5.40), broader bony interorbital space (3.43–3.57 in head, compared with 4.44–4.63), and longer third anal-fin spine (1.85–2.50, compared with 2.55–3.19). *Tosanoides flavofasciatus* additionally differs from *Tosanoides obama* in having a deeper body (2.29-2.69 in SL, compared with 2.84–2.88).

The three species can also be easily distinguished from each other on the basis of life color.

Based on the five observed individuals of *Tosanoides obama*, none of which were larger than the holotype, this species appears to be much smaller than the other two, adults of which are typically 55–85 mm SL.

#### Genetic comparisons.

Vertebrate mtDNA barcode (cytochrome oxidase I) sequences obtained from the holotype and paratype of *Tosanoides obama* reveal 9.5–10% uncorrected sequence divergence when compared with the other two described species of *Tosanoides*. This is somewhat higher than many species-level divergences in other fish taxa (e.g., Johns and Avise 1998, Bellwood et al. 2004, Fessler and Westneat 2007, Randall and Rocha 2009, Rocha 2004, Rocha et al. 2008, Pyle and Kosaki 2016). The accepted mtDNA clock rate of approximately 2% per million years in fishes (Bowen et al. 2001, Reece et al. 2010) indicates divergence between these species on the order of 5 million years. Nevertheless, based on a preliminary genetic analysis, *Tosanoides obama* has closer genetic affinities to both *Tosanoides* species than to representatives of eight other Indo-Pacific anthiadine genera (including *Luzonichthys* Herre, 1936, *Nemanthias* Smith, 1954, *Odontanthias* Bleeker, 1873, *Plectranthias* Fowler, 1935, *Pseudanthias* Bleeker, 1871, *Sacura* Jordan & Richardson, 1910, *Serranocirrhitus* Watanabe, 1949, and *Tosana* Smith & Pope, 1906). On this basis, as well as morphological comparisons, we are confident in assigning the new species to the genus *Tosanoides* until a more exhaustive investigation of phylogenetic relationships among the species in this subfamily is completed.

#### Discussion.

*Tosanoides obama* is another example of several new fish species that have been discovered on deep coral reefs over the past several decades, mostly involving the use of modern mixed-gas closed-circuit rebreather diving technology (Pyle 1996, 2000). In recent years there has been increased attention focused on mesophotic coral ecosystems (MCEs), coral-reef habitat at depths of approximately 30–150 m in tropical regions worldwide (Hinderstein et al. 2010, Baker et al. 2016). Many more new species of fishes and other reef-associated marine organisms are likely to be discovered as a result of continued exploratory work in this poorly documented environment.

The fish subfamily Anthiinae (Anthiadides Poey, 1861, type genus *Anthias* Bloch, 1792, stem *Anthi*-), is a homonym of the beetle subfamily Anthiinae (Anthies Bonelli, 1813, type genus *Anthia* Weber, 1801, stem *Anthi*-). According to Article 55.3 of the International Code of Zoological Nomenclature (ICZN 1999), homonymous family-group names in current use based on similar (but not identical) genus-group names must be referred to the Commission for a ruling to remove homonymy. A case is currently in preparation to formally resolve this homonymy through application to the ICZN. Until an Opinion is issued, we follow van der Laan et al. (2014, 2016) and Carvalho-Filho (2016) in using the spelling “Anthiadinae” to represent the subfamily for this new species, instead of the more commonly used (but homonymous) spelling “Anthiinae”.

## Supplementary Material

XML Treatment for Tosanoides obama
